# Nutritional anti-nutritional chemical composition and antioxidant activities of the leaves of the sea cliff dwelling species *Limonium spathulatum* (Desf.) Kuntze

**DOI:** 10.3389/fpls.2022.979343

**Published:** 2022-11-15

**Authors:** Seria Youssef, Luísa Custódio, Maria João Rodrigues, Catarina G. Pereira, Ricardo C. Calhelha, José Pinela, Lillian Barros, József Jekő, Zoltán Cziáky, Karim Ben Hamed

**Affiliations:** ^1^ Laboratory of Extremophile Plants, Center of Biotechnology of Borj Cedria, Hammam-Lif, Tunisia; ^2^ Centre of Marine Sciences, Universidade do Algarve, Faro, Portugal; ^3^ Centro de Investigação de Montanha (CIMO), Instituto Politécnico de Bragança, Bragança, Portugal; ^4^ Agricultural and Molecular Research and Service Institute, University of Nyíregyháza, Nyíregyháza, Hungary

**Keywords:** antinutritional, nutritional, phenolic compounds, sea lavender, antioxidant activity

## Abstract

This work explored the nutritional and antioxidant properties of the leaves of the halophytic species *Limonium spathulatum* (Desf.) Kuntze from Tunisian sea cliffs. Furthermore, the analysis of the total phenolics and flavonoids contents and their individual compounds using high-performance liquid chromatography coupled with electrospray ionization mass spectrometry (HPLC-ESI-MS/MS) were also studied. *L. spathulatum* leaves had high levels of moisture, ash, neutral detergent fiber, and acid detergent fiber, but low concentrations of crude protein, crude fat and acid detergent lignin. It contained low carbohydrates levels, and low energetic values. The most abundant macroelements were Cl, Na and Ca while the microelements detected in the highest levels were Fe and Zn. No relevant α-amylase inhibition was observed, and no toxic metals (Pb and Cd) and phytic acid were detected. The ethanol and the hydroethanolic extracts had the highest capacity to scavenge free radicals, to chelate iron and copper and to inhibit lipid peroxidation. The same samples were also the most active towards oxidative haemolysis. These extracts contained high total phenolic and flavonoid contents. HPLC analysis, performed on ethanolic extracts identified 58 individual compounds known for their high antioxidant actvitiy including hydroxybenzoic acids (gallic, syringic acids), hydroxycinnamic acids (caffeic, coumaric, ferulic acids) and flavonoids (catechin, epigallocatechin gallate and naringin).In conclusion, the leaves of Tunisian accession of *L. spathulatum* were good source of minerals and fibers useful in the human diet for attaining nutritional sufficiency. The high *in vitro* and *ex vitro* antioxidant activities associated with high favonoids contents and compounds suggest the possibility to use the extracts of *L. spathulatum* in herbal products with the aim of improving general health and well-being, and/or as food additives for preventing lipid oxidation of lipid-rich foods.

## 1 Introduction

The Mediterranean basin is considered one of the world’s biodiversity hotspots due to its high variety of plant species and endemism’s Petropoulos et al., 2018; Bolaric et al., 2021; Hasanbegovic et al., 2021; Curadi et al., 2022)

The *Limonium* genus (Plumbaginaceae) includes approximately 370 species of perennial herbs and shrubs belonging to a particular type of halophytes,’recretohalophytes’, that can secrete salt from their leaves through salt bladders and salt glands, as a mechanism of adaptation to high salinity conditions (Yuan et al., 2016; González-Orenga et al., 2021).

*Limonium* species commonly known as sea lavenders are widely distributed in the Mediterranean region, mainly in the North-Eastern and Southern countries (Brullo, 1978; Brullo, 1980; Brullo and Erben, 1989; Brullo and Erben, 2016). In North Africa were identified 107 species, and from these, 26 are endemic to Tunisia (Dobignard et al., 2013). Some species are highly valued as ornamental plants (*e.g*., *L. sinuatum* (L.) Mill., and *L. latifolium* (Sm.) Kuntze, *L. perezii* (Stapf) F.T. Hubb. (Morgan and Funnell, 2018; González-Orenga et al., 2021). Other species have ethnopharmacological uses against several ailments, including cardiovascular and inflammatory conditions, (Aniya et al., 2002; Murray et al., 2004; González-Orenga et al., 2021), are rich in bioactive polyphenolic compounds, in particular flavonoids (Lin and Chou, 2000; Ye and Huang, 2006; Geng et al., 2015), and display several functional properties, such as antioxidant, anti-inflammatory and immunomodulation (Kandil et al., 2000; Aniya et al., 2002; Kuo et al., 2002; Mahasneh, 2002; Murray et al., 2004; Cantrell et al., 2007; Smirnova et al., 2009; Lee et al., 2011; Nostro et al., 2012; Tang et al., 2012; Saidana et al., 2013; Ali et al., 2013; Rodrigues et al., 2015; Souid et al., 2019).

**Figure 1:**
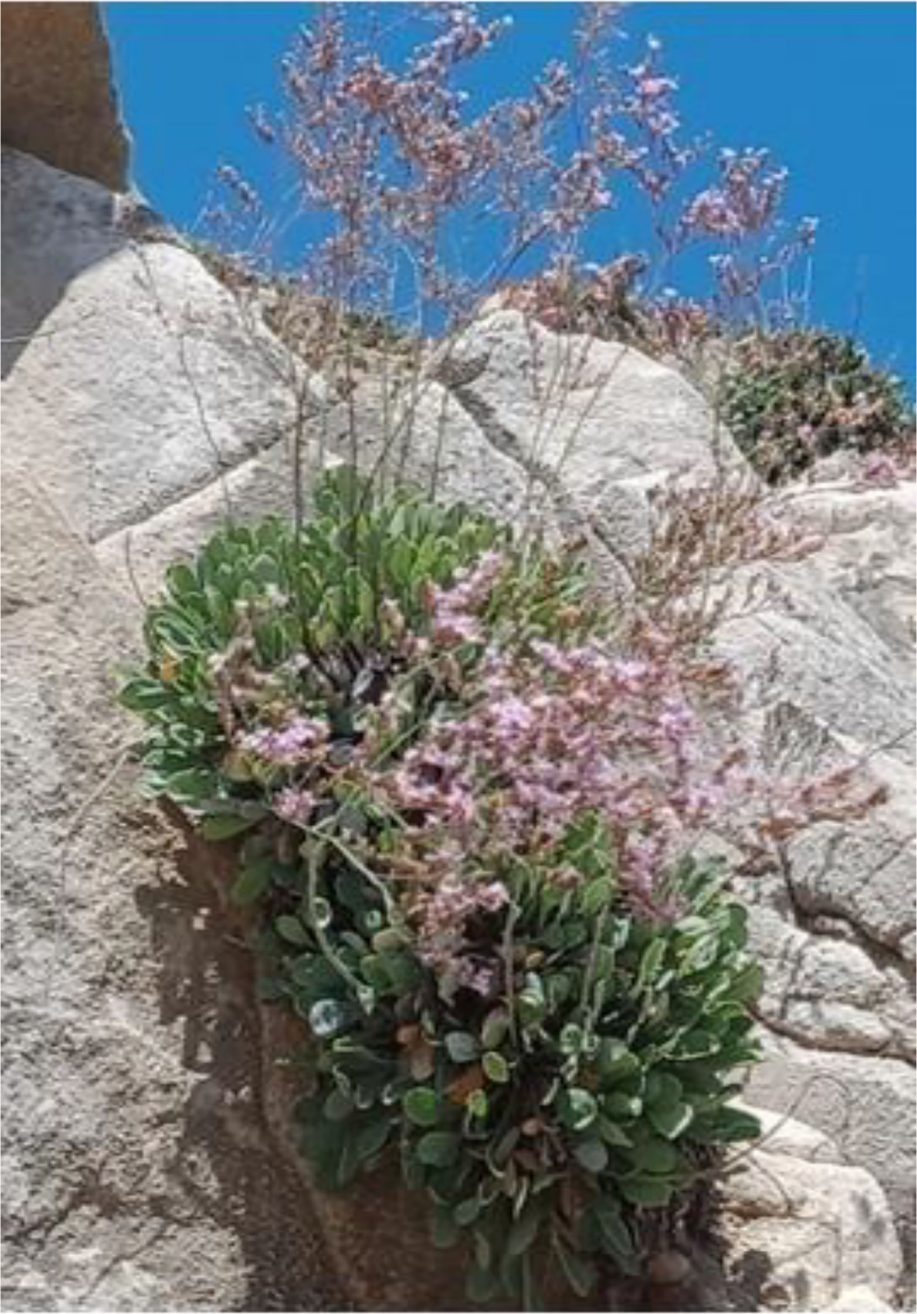
General aspects of *Limonium spathulatum, Tabarka rocky coast (Tunisia)*, 36°57’23” N8°45’28.5” E (Photo by Seria Youssef, 2019).

Having in mind the high importance of single- country endemic plants as sources of high added value products (Shelef et al., 2017; Sefi et al., 2021), this work focused on the species *L. spathulatum* (Desf.) kuntze which grow wild in the sea cliffs of Tunisia (**Figure 1**). Despite the traditional uses and potential commercial applications of several *Limonium* species, information regarding *L. spathulatum* is limited and refers to the phenolic composition and antioxidant, anti-alzheimer, anti-diabetic, and anti-inflammatory *in vitro* properties of organic extracts extracts from aerial parts collected from plants in Algeria (Mazouz et al., 2020), mineral, phenolic, carotenoids and vitamins contents, *in vitro* antioxidant properties, erythrocytes cellular antioxidant activity (CAA-RBC) and oxidative hemolysis protection of methanol extracts from plants collected in Tunisia (Souid et al., 2019).

This work aimed to explore the use of the coastal *L. spathulatum* leaves in the food industry either as food and as a source of bioactive herbal products. For that purpose, leaves were collected in Tunisian sea cliffs and profiled firstly for their nutritional and anti-nutritional properties. The *in vitro* and *ex vivo* antioxidant properties and the total levels of phenolics and flavonoids of food grade leaf extracts were also determined. Furthermore, analysis of individual phenolics and flavonoids compounds was carried out by HPLC-ESI-MS/MS.

## 2 Material and methods

### 2.1 Chemicals

The chemicals used in this work were all analytical grade. Ethylenediamine tetraacetic acid (EDTA) was purchased from Fluka (Steinheim, Germany),while copper sulfate pentahydrate (CuSO_4_.5 H_2_O), and ferrozine were acquired from Merck (Darmstadt, Germany).Butylated hydroxytoluene (BHT), quercetin, 1,1-diphenyl-2-picrylhydrazyl (DPPH), rutin hydrate, 2,2-azino-bis(3-ethylbenzothiazoline-6-sulfonic acid) (ABTS) radicals, phosphoric acid, and pyrocatechol violet (PV). Phosphate buffered saline (PBS), trolox (6-hydroxy-2,5,7,8-tetramethylchroman-2-carboxylic acid), thiobarbituric acid (TBA), 2.2’-azobis (2-methylpropionamidine) dihydrochloride (AAPH), sulforhodamine B, and ellipticine were purchased from Sigma-Aldrich (St. Louis, MO, USA). Ethanol was purchased from Riedel de Haën (Buchs, Switzerland). Additional reagents and solvents were obtained from VWR International (Leuven, Belgium).

### 2.2 Plant material and extracts preparation

Leaves of *L. spathulatum* were collected in March of 2019 from flowering adult plants growing in coastal areas of Tabarka in Tunis (Tunisia) (coordinates: 36°57’23” N 8°45’28.5” E). The taxonomical classification was performed by the botanist Dr. Abderrazek Smaoui (Center of Biotechnology of Borj Cedria, Tunisia) and a voucher specimen is kept in the herbarium of the Laboratory of Extremophile Plants (voucher code LPEH01). Depending on the analysis, two drying methods were used. For the nutritional analysis, samples were lyophilized, ground in liquid nitrogen, and stored at -20°C. For the preparation of the extracts, leaves were dried at 37°C for one week, milled and stored in the dark at 4°C. For extract’s preparation, dried powder was mixed with ethanol (100 % and 50 %, w/w) and water (1:40, w/w), and extracted overnight, at room temperature (RT) with stirring. The extracts were then filtered (Whatman paper no. 4), and dried in a rotary evaporator under reduced pressure at 40 °C. The water extracts were freeze dried. The resulting dried extracts were weighed, dissolved in the corresponding solvent at the concentration of 50 mg/mL, and stored at −20°C until analysis.

### 2.3 Nutritional properties

#### 2.3.1 Proximate composition

Moisture was determined as the difference of the weight of the fresh leaves before and after drying at 90°C for 2 d. Ash was determined by incineration of dried biomass at 500°C in a muffle furnace for 7 h. Crude protein content was estimated by the Kjeldahl method and was obtained by multiplying by 6.25 the evaluated nitrogen. Crude fat was determined by a modified protocol of the Bligh and Dyer method (Bligh and Dyer, 1959). Total sugar content was determined using the Anthrone method of Yemm and Willis (1954), while neutral detergent fibre (NDF), acid detergent fibre (ADF) and acid detergent lignin (ADL) were determined in agreement with the International Organization for Standardization (ISO) directives 16472:2006, 13906:2008 and 13906:2008, respectively). Metabolizable energy (ME) was calculated using the Atwater specific factor for vegetables (FAO, 2003) according to the following equation: ME (kcal) = 2.44 × (g protein) + 3.57 × (g carbohydrate) + 8.37 × (g lipid).

#### 2.3.2 Minerals

Dried leaf samples were ground into fine powder. 10 mg of leaf powder were mixed in sulfuric acid (H_2_SO_4_, 1N) for 1 h at 80°C to extract the different minerals (Zorrig et al., 2010). The extract samples were prepared by filtration with a 0.45 µm pre-syringe filter. Sodium (Na), potassium (K) and calcium (Ca) were assayed by flame emission photometry. Iron (Fe), zinc (Zn), magnesium (Mg), cadmium (Cd) and lead (Pb) were determined through atomic absorption spectrophotometry. Different standard solutions were used : 0-20 µg/ml for Na, K, Ca, Mg and Fe, 0-2 µg/ml for Zn, Cd and Pb, Phosphorous (P) was measured by spectrophotometry at 430 nm. Chloride (Cl) was determined by chloride analyzer model 926. Iodine determination was performed according to the European Standard EN 15111:2007. Briefly, dried samples (approximately 100 mg) were weighed directly in borosilicate glass tubes (16×125 mm) to which ultrapure water (> 18.2 MΩ. cm at 25°C) and TMAH (25 wt. % in H_2_O) were added. The glass tubes were capped and placed in a drying oven adjusted to 90 ± 3°C. Iodine was analyzed by inductively coupled plasma mass spectrometry (ICP-MS) using an iCAP^TM^ Q instrument (Thermo Fisher Scientific, Bremen, Germany). The elemental isotope ^127^I was monitored for quantitative purposes. The elemental isotope ^125^Te was used as internal standard (IS).

### 2.4 Anti-nutritional properties and toxic factors

Trypsin inhibition was assessed by the method of (Bacon et al., 1995) adapted to 96-well microplates. In brief, samples (60 µL at 1 mg/mL), were mixed with the enzyme (60 µL; 0.02 mg/mL of bovine in 0.001 M of HCL) and incubated in the dark, for 15 min at 41°C. Then, 150 µL of the substrate solution (BAPNA in 20 mM CaCl_2_ and 50 mM Tris-HCl pH 8.2), were added and incubated for 10 min, at RT. The reaction was stopped by adding 30 µL of 30 % acetic acid, and the absorbance was measured at 410 nm. Results were expressed as inhibition (%) relative to a blank containing the solvent of the extraction. Inhibition towards α-amylase was evaluated by the method described by (Xiao et al., 2006) using extracts at the concentration ranging from 0.009 to 5 mg/mL. The results were expressed as inhibition (%) relative to a blank containing the solvent of the extraction. The phytic acid content of the extracts was determined according to the protocol described by (Lorenz et al., 2007), in extracts at the concentration of 150 mg/mL. Results were calculated in relation to a calibration curve made with different concentrations of phytic acid.

### 2.5 Determination of *in vitro* antioxidant activity by radical based methods

The radical scavenging activity (RSA) of the extracts was tested towards DPPH and ABTS according to the methods described previously (Rodrigues et al., 2015). Leaf samples (22 µL, at concentrations ranging from 0.009 to 5 mg/mL) were mixed with 200 µL of DPPH solution (120 µM) in methanol in 96-well microplates, and incubated in darkness at RT for 30 min. The absorbance was measured at 517 nm (EZ read 400, Biochrom). For RSA determination on ABTS radical, a stock solution of ABTS•+ (7.4 mM) was diluted with ethanol to obtain an absorbance of at least 0.7 at 734 nm (EZ read 400, Biochrom). The samples (10 µL at concentrations between 0.009 and 5 mg/mL) were mixed in 96-well microplates with 190 µL of ABTS•+ solution. After an incubation for 6 min, the absorbance was measured at 734 nm (EZ read 400, Biochrom). RSA was expressed as percentage relative to the negative control containing the corresponding solvent, and as half-maximal effective concentration (EC_50_ values, mg/mL) when possible. Butylated hydroxytoluene (BHT) was used as a positive control at concentrations up to 1 mg/mL.

### 2.6 Determination of *in vitro* antioxidant activity by metal-based methods

The ferric reducing antioxidant power (FRAP), the metal chelating activity on copper (CCA) and iron (ICA) were determined according to previously described protocols (Rodrigues et al., 2015). FRAP determines the ability of the extracts to reduce Fe3+. Samples (50 µL at concentrations from 0.009 to 5 mg/mL), distilled water (50 µL) and 1% potassium ferricyanide (50 µL) were mixed and incubated at 50 °C for 20 min. Then, 50 µL of 10% trichloroacetic acid (w/v) and ferric chloride solution (0.1 %, w/v) were added, and absorbance was measured at 700 nm (EZ read 400, Biochrom).

The CCA estimates the ability of the extracts to chelate Cu^2+^. 30 µl of samples (30 µL at concentrations ranging from 0.009 to 5 mg/mL), 200 µL of Na acetate buffer 50 mM (pH 6), 6 µL of pyrocatechol violet (4 mM) dissolved in Na acetate buffer, and 100 µL of CuSO_4_ 5H_2_0 (50 µg/mL in water) were mixed in 96-well microplates. Aborbance was measured at 632 nm using a microplate reader (EZ read 400, Biochrom). BHT (1 mg/mL) was used as a positive control.

The ICA chelating activity was determined by measuring the formation of the Fe^2+^ ferrozine complex according to (Rodrigues et al., 2015). 30 µl of the samples were mixed with 200 µL of dH_2_0 and 30 µL of a FeCl_2_ solution (0.1 mg/mL in water) in 96-well microplates. After 30 min, 12.5 µL of ferrozine solution (40 mM in water) was added. Aborbance was measured at 562 nm using a microplate reader (EZ read 400, Biochrom).

EDTA (1 mg/ml) was used as the positive control.

For all the above mentionned methods, increased absorbance of the reaction mixture indicated increased reducing power. Results were expressed as (%) of inhibition, relative to the positive control, (FRAP) and to the negative control (CCA and ICA) and as EC_50_ values.

### 2.7 Determination of *ex vivo* antioxidant activity

The *ex vivo* antioxidant activity of the extracts were evaluated by their ability to inhibit lipid peroxidation of porcine brain cells by the thiobarbituric acid reactive substances (TBARS) assay, and by the oxidative haemolysis inhibition assay (OxHLIA), using a sheep erythrocyte solution and AAPH as a free radical generator, according to the methods described in (Rodrigues et al., 2021). For TBARS assay, a porcine brain cell solution (1:2, w/v; 100 µL) was incubated with 200 µL of sample or trolox, 100 µL of FeSO4 (10 µM) and 100 µL of ascorbic acid (0.1 mM) at 37°C for 1 h. Then, 500 µL of trichloroacetic acid (28 % w/v) and 380 µL of thiobarbituric acid (TBA; 2 % w/v) were added and the mixture was heated at 80 °C for 20 min. After centrifugation, the color intensity of the malondialdehyde (MDA)-TBA complexes formed in the system was measured at 532 nm.

For OxHLIA, a sheep erythrocyte solution (2.8 %, v/v; 200 µL) prepared in phosphate-buffered saline (PBS, pH 7.4) was mixed with 400 µL of either: Sample, PBS, distilled water or trolox. After pre-incubation at 37°C for 10 min with shaking, 200 µL of AAPH (160 mM) were added and absorbance was measured kinetically at 690 nm (EZ read 400, Biochrom) until complete haemolysis. The extracts were tested at concentrations ranging from 0.0625 to 2 mg/mL, and trolox (3.125 – 100 µg/mL) was used as the positive control. Results were expressed as EC_50_ values (µg/mL), considering a 60 min Δ*t* in OxHLIA.

### 2.8 Total phenolic (TPC) and flavonoid (TFC) contents

The TPC and TFC were determined in the extracts at the concentration of 5 mg/mL. TPC was determined by the Folin-Ciocalteu (F-C) assay, and TFC by the aluminum chloride colorimetric method adapted to 96-well microplates. In brief, the extracts (5 µl at a concentration of 5 mg/ml) were mixed with 100 µl of tenfold diluted F-C reagent and incubated at RT for 10 min. Subsequently, 100 µ l of Na_2_ CO_3_ (75 g/1, w/v) were added and the absorbance was measured on a microplate reader (EZ read 400, Biochrom) at 725 nm after a 90 min incubation period at RT. TPC was expressed as gallic acid equivalents (GAE) in milligrams per gram of dry extract using a calibration curve plotted from gallic acid standard solutions (0 – 2 mg ml -1).

The total flavonoid content (TFC) of the extracts was estimated by the aluminium chloride (AlCl_3_) colorimetric method according to (Akrout et al., 2011). 1 ml of diluted sample was mixed with 1 ml of 2% aluminium trichloride (AlCl_3_) methanolic solution. After incubation at room temperature for 15 min, the absorbance of the reaction mixture was measured at 430 nm with a microplate reader (EZ read 400, Biochrom). Results were expressed as milligrams of quercetin equivalents per gram of dried sample (mg QE/g DW) using a calibration curve produced with quercetin concentrations between 0.01 and 2.5 mg/mL.

### 2.9 High-performance liquid chromatography coupled with electrospray ionization mass spectrometry (HPLC-ESI-MS/MS) analysis of phenolic and flavonoid compounds.

The chemical composition of the extracts was determined using a Dionex Ultimate 3000RS UHPLC instrument. Samples were filtered (0.22 μm PTFE filter membrane, Labex Ltd, Hungary) before HPLC analysis, and injected onto a Thermo Accucore C18 (100 mm x 2.1, mm i. d., 2.6 μm) column thermostated at 25 °C (± 1 °C). The solvents used were water (A) and methanol (B), acidified with 0.1% formic acid, and the flow rate was maintained at 0.2 mL/min. A gradient elution was used: 5% B (0–3 min), a linear gradient increasing from 5% B to 100% (3–43 min), 100% B (43–61 min), a linear gradient decreasing from 100% B to 5% (61–62 min) and 5% B (62–70min). The column was coupled with a Thermo Q-Exactive Orbitrap mass spectrometer (Thermo Scientific, USA) equipped with electrospray ionization source. Spectra were recorded in positive and negative-ion mode, respectively. The trace finder 3.1 (Thermo Scientific, USA) software was applied for target screening. Most of the compounds were identified based on previously published work or data found in the literature. The exact molecular mass, isotopic pattern, characteristic fragment ions and retention time were always used to identify the molecules.

### 2.10 Statistical analysis

Experiments were conducted at least in triplicate and results were expressed as mean ± standard deviation (SD). Differences in significance (*p*< 0.05) were evaluated by one-way analysis of variance (ANOVA), pursued by the Tukey HSD test. Statistical analyses were performed using XLStat2014®. The EC_50_ values were determined by sigmoidal fitting of the data in the GraphPad Prism v. 5.0 software.

## 3 Results

### 3.1 Nutritional and anti-nutritional properties

The proximate composition (moisture, crude protein, crude fat, carbohydrates, metabolizable energy), fiber (NDF, ADF, ADL) and iodine were determined in *L. spathulatum* leaves and results are summarized in **Table 1**. *Limonium spathulatum* had high levels of moisture (77.7 %), ash (7.10 %), NDF (35.7 %), and ADF (25.5 %), but low concentrations of crude protein (9.93 %), crude fat (0.36 %) and ADL (12.6 %). *Limonium spathulatum* also had a low carbohydrates level (1.79%), and a low energetic value (33.7 kcal/100 g, dw). The iodine level of *L. spathulatum* was 0.629 mg/Kg (dw). Minerals were also determined, and results are depicted in **Table 2**. The most abundant macroelements were Cl^-^ (42.4 mg/g, dw), Ca (7.1 mg/g, dw) and Na (16.2 mg/g, dw), while the microelements detected in the highest levels were Fe (422 µg/g,dw) and Zn, (25.3 µg/g,dw). The toxic elements Pb and Cd were not detected.

**Table 1:** Nutritional profile of leaves of *Limonium spathulatum*.

Proximate composition	Value
Moisture (%)	77.7 ± 0.53
Ash (%)	7.10 ± 0.06
Crude protein (%)	9.93 ± 0.11
Crude fat (%)	0.36 ± 0.08
Carbohydrates (%)	1.79 ± 0.06
Metabolizable energy (kcal/100 g DW)	33.6
Neutral detergent fibre (NDF) (%)	35.7 ± 2.64
Acid detergent fiber (ADF) (%)	25.5 ± 1.66
Acid detergent lignin (ADL) (%)	12.6 ± 1.25

Values represent the mean ± SD of at least three repetitions (n = 3).

DW, dry weight; nd, not detected.

**Table 2:** Mineral composition of leaves of *Limonium spathulatum*.

Macroelements	mg/g DW	mg/100 g FW
Sodium (Na)	16.20 ± 1.08	361.60
Potassium (K)	9.18 ± 0.82	204.90
Magnesium (Mg)	10.50 ± 0.56	234.30
Calcium (Ca)	17.10 ± 1.52	381.60
Chloride (Cl)	42.40 ± 1.28	946.40
Phosporous (P)	2.28 ± 0.21	50.80
**Microelements**	µg/g DW	mg/100 g FW
Iron (Fe)	422 ± 2.51	9.41
Zinc (Zn)	25.30 ± 1.86	0.57
Copper (Cu)	12.20 ± 0.06	0.27
Iodine (I)	0.62 ± 0.04	0.14
Cadmium (Cd)	nd	Nd
Lead (Pb)	nd	Nd

Values represent the mean ± SD of at least three repetitions (n = 3).dw, dry weight; nd, not detected.

The presence of antinutritional and toxic factors in the extracts was evaluated in terms of trypsin and amylase inhibition, and levels of phytic acid (**Table 3**). A high trypsin inhibition was observed with the water extract (82.8%), followed by the hydroethanolic (75.1%) and ethanol (72%) extracts. No relevant α-amylase inhibition was observed, and no phytic acid was detected.

**Table 3:** Antinutrients and toxic factors in leaves of *Limonium spathulatum*.

Extract	Trypsin inhibition (%)	α-Amylase inhibition(%)	Phytic acid
Ethanol	72.0 ± 1.80	9.37 ± 1.7	Nd
Water	82.8 ± 1.63	10.2 ± 1.13	Nd
Hydroethanolic	75.1 ± 1.11	29.6 ± 1.22	Nd

Values represent the mean ± SD of at least three repetitions (n = 3).

The extracts were tested for trypsin and amylase inhibition at 1 mg/mL, for phytic acid quantification up to 150 mg/mL.

nd, not detected.

### 3.2 Antioxidant properties

The antioxidant potential of the extracts was evaluated by five *in vitro* methods, namely two radical-based assays (RSA on DPPH and ABTS radicals), and three metal-related methods (FRAP and metal chelation of iron and copper). As can be seen in **Table 4**, the ethanol and the hydroethanolic extract had the highest capacity to scavenge free radicals, with EC_50_ values of 0.04 and 0.08 mg/mL for DPPH and 0.10 and 0.05 mg/mL for ABTS, respectively. For those extracts, the EC_50_ values were similar or even lower than those obtained with the positive control (BHT, 0.11 and 0.141 mg/mL for the DPPH and ABTS assays, respectively). Samples had no capacity to chelate iron, but exhibited significant copper chelating properties, and again, the best results were obtained with the ethanol and hydroethanolic extracts, with similar EC_50_ values (0.48 mg/mL). Samples also had the capacity to chelate iron, with the ethanol and hydroethanolic samples exhibiting the lowest EC_50_value (0.04 mg/mL).

**Table 4:** Radical scavenging activity (RSA) on DPPH and ABTS, metal chelating activity on copper (CCA) and iron (ICA) and ferric reducing activity power (FRAP) of different extracts of Limonium *spathulatum*.

Sample	DPPH	ABTS	ICA	CCA	FRAP
Ethanol	0.04 ± 0.00^a^	0.10 ± 0.01^a^	Nr	0.48 ± 0.02^b^	0.04 ± 0.00^a^
Water	0.32 ± 0.01^c^	0.15 ± 0.03^a^	Nr	0.56 ± 0.07^b^	0.09 ± 0.00^b^
Hydroethanolic	0.08 ± 0.00^ab^	0.05 ± 0.01^a^	Nr	0.48 ± 0.02^b^	0.04 ± 0.00^a^
Positive controls					
BHT*	0.11 ± 0.00^b^	0.141 ± 0.00^a^	Nt	nt	nt
EDTA*	nt	Nt	0.06 ± 0.00	0.17 ± 0.00^a^	nt

Results are expressed as effective maximal inhibitory concentration (EC_50_) values in mg/mL.

Values represent the mean ± SD of at least three experiments performed in triplicate (n = 9). Comparison was made between extract, for the same assay, and values followed by letters are significantly different referring to the Tukey HSD test (p < 0.05).

DPPH, 2, 2-diphenyl-1-picrylhydrazyl; ABTS, 2, 2′-azino-bis (3-ethylbenzothiazoline-6-sulfonic acid) diammonium salt.

nr, the EC_50_ value was not reached.

nt, not tested.

*Butylated hydroxytoluene (BHT, E320) and ethylenediaminetetraacetic acid (EDTA), positive control.

**Figure 2:**
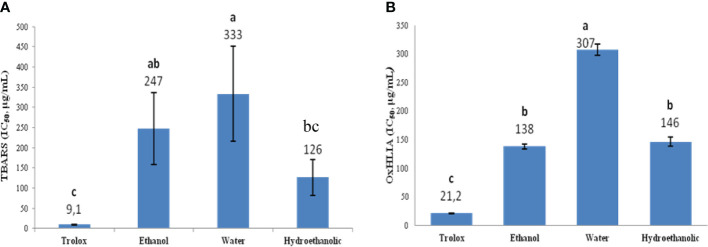
*Ex vivo* antioxidant activity (**A**: TBARS, **B**: OxHLIA), of ethanol, water and hydroethanolic extracts of *Limonium spathulatum*. Values represent the mean ± SD of at least three repetitions (n = 3). For each assay bars marked with different letters are significantly different at *p< 0.05* (Tukey HSD test).

To gain further knowledge on the antioxidant properties of the extracts, samples were tested by two *ex vivo* antioxidant assays, which allowed to evaluate their capacity to inhibit lipid peroxidation (by the TBARS formation) and oxidative haemolysis (OxHLIA) (**Figure 2**). The hydroethanolic and the ethanol extracts displayed the highest capacity to inhibit lipid peroxidation, with EC_50_ values of 126 and 247 μg/mL, respectively. The same samples were also the most active towards oxidative haemolysis, with EC_50_ values of 138 and 146 μg/mL for the ethanol and the hydroethanolic extract, respectively.

### 3.3 Total phenolic and flavonoid quantification and HPLC identification

**Figure 3:**
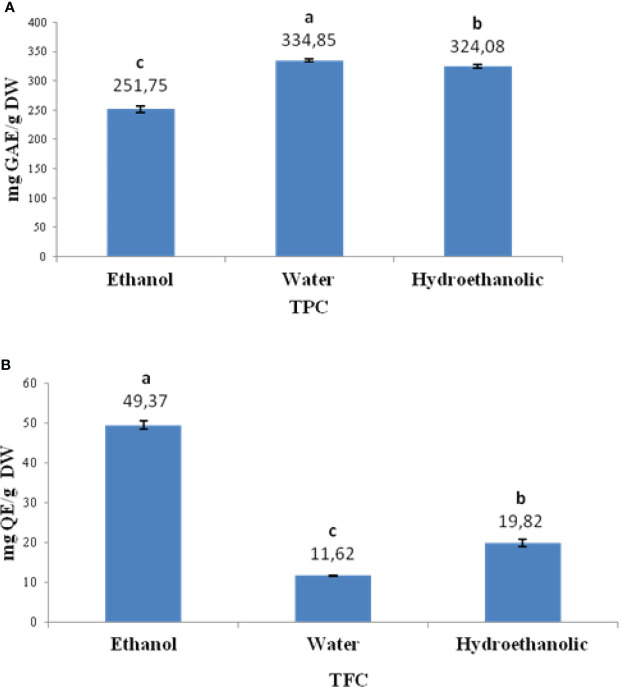
Total levels of phenolics (TPC) **(A)** and flavonoids (TFC) **(B)** of ethanol, water and hydroethanolic extracts of *Limonium spathulatum*. Values represent the mean ± standard deviation (SD) of at least six repetitions (n = 6), expressed as equivalents GAE/g for TPC and QE/g for TFC, DW For each group of compounds, bars marked with different letters are significantly different at *p*< 0.05 (Tukey HSD test).

The total levels of phenolics (TPC) and flavonoids (TFC) were quantified in the extracts, and results are shown in (**Figure 3**). The TPC peaked in the water (334.85 mg GAE/g, dw) and hydroethanolic extracts (324.0 mg GAE/g, dw), followed by the ethanol extract (251.7 mg GAE/g, dw). In the contrary, the ethanol extract had the highest level of flavonoids (49.3 mg QE/g,), followed by the hydroethanolic (19.8 mg GAE/g, dw) and the water (11.6 mg GAE/g, dw) extracts.

To gain a deeper knowledge on the individual chemical components of the extracts, an analysis was made by HPLC-ESI-MS/MS, and results are summarized in **Table 5**. The ethanolic extract was used for this HPLC analysis because of its high antioxidant activities. HPLC analysis identified 58 individual compounds (**Table 5**) including mainly hydroxybenzoic acids (gallic, syringic acids), hydroxycinnamic acids (caffeic, coumaric, ferulic acids) and flavonoids (catechin, epigallocatechin gallate and naringin).

**Table 5:** High-performance liquid chromatography coupled with electrospray ionization mass spectrometry (HPLC-ESI-MS/MS) tentative identification of metabolites present in the ethanolic extracts of *Limonium spathulatum*.

	Formula	RT	[M + H]+	[M – H]-
Quinic acid	C7H12O6	2,11		191,05557
Shikimic acid	C7H10O5	2,16		173,04500
Galloylhexose	C13H16O10	2,87		331,06653
Gallic acid (3,4,5-Trihydroxybenzoic acid)	C7H6O5	3,18		169,01370
Gallocatechin (Gallocatechol)	C15H14O7	5,63		305,06613
Coumaroylhexose sulfate isomer 1	C15H18O11S	7,79		405,04916
Caffeoylhexose sulfate isomer 1	C15H18O12S	9,00		421,04408
Uralenneoside or isomer	C12H14O8	11,03		285,06105
Caffeoylhexose	C15H18O9	11,81		341,08726
Coumaroylhexose sulfate isomer 2	C15H18O11S	12,22		405,04916
Caffeoylhexose sulfate isomer 2	C15H18O12S	12,80		421,04408
Epigallocatechin (Epigallocatechol)	C15H14O7	13,45		305,06613
Chlorogenicacid (3-O-Caffeoylquinic acid)	C16H18O9	14,42	355,10291	
Coumaroylhexose isomer 1	C15H18O8	14,46		325,09235
Caffeic acid	C9H8O4	14,60		179,03444
Biflorin	C16H18O9	14,78	355,10291	
Digalloylhexose	C20H20O14	14,98		483,07749
Coumaroylhexose isomer 2	C15H18O8	15,16		325,09235
Isobiflorin	C16H18O9	15,56	355,10291	
Epigallocatechin-3-O-gallate (Teatannin II)	C22H18O11	16,25		457,07709
Dihydrokaempferol-O-hexoside	C21H22O11	17,18		449,10839
4-Coumaric acid	C9H8O3	17,99		163,03952
Coumaroyl-hexosylglycerate	C18H22O11	18,09		413,10839
Isololiolide	C11H16O3	18,63	197,11777	
Ferulic acid	C10H10O4	19,36		193,05009
Unidentified alkaloid	C13H12N2O3	19,55	245,09262	
Loliolide	C11H16O3	19,84	197,11777	
Myricetin-O-hexoside	C21H20O13	20,37		479,08257
Myricetin-3-O-rutinoside	C27H30O17	21,05		625,14048
Myricetin-O-pentoside	C20H18O12	21,50		449,07201
Myricitrin (Myricetin-3-O-rhamnoside)	C21H20O12	21,68		463,08765
N-cis-Feruloyltyramine	C18H19NO4	22,35	314,13924	
Hyperoside or Isoquercitrin	C21H20O12	22,31		463,08765
Rutin (Quercetin-3-O-rutinoside)	C27H30O16	22,60		609,14557
Coatline A or isomer	C21H24O10	22,74		435,12913
Methoxy-pentahydroxy(iso)flavone-O-hexoside	C22H22O13	22,87		493,09822
Myricetin (3,3’,4’,5,5’,7-Hexahydroxyflavone)	C15H10O8	23,80		317,02974
Kaempferol-7-O-glucoside	C21H20O11	23,84		447,09274
Phlorizin	C21H24O10	24,05		435,12913
Quercitrin (Quercetin-3-O-rhamnoside)	C21H20O11	24,21		447,09274
Astragalin (Kaempferol-3-O-glucoside)	C21H20O11	24,41		447,09274
Kaempferol-3-O-rutinoside (Nicotiflorin)	C27H30O15	24,54		593,15065
N-trans-Feruloyltyramine	C18H19NO4	24,60	314,13924	
Dimethoxy-tetrahydroxy(iso)flavone isomer 1	C17H14O8	25,79		345,06104
Afzelin (Kaempferol-3-O-rhamnoside)	C21H20O10	26,19		431,09782
Dihydroactinidiolide	C11H16O2	26,66	345,09743	
Quercetin (3,3’,4’,5,7-Pentahydroxyflavone)	C15H10O7	26,71		301,03483
Naringenin (4’,5,7-Trihydroxyflavanone)	C15H12O5	27,23		271,06065
Quercetin-3-O-methyl ether	C16H12O7	28,10		315,05048
Phloretin (Dihydronaringenin)	C15H14O5	28,23		273,07630
Dimethoxy-tetrahydroxy(iso)flavone isomer 2	C17H14O8	28,34		345,06104
Trihydroxy-trimethoxy(iso)flavone isomer 1	C18H16O8	30,37		359,07670
Trihydroxy-trimethoxy(iso)flavone isomer 2	C18H16O8	31,10		359,07670
Malyngic acid or isomer	C18H32O5	32,30		327,21715
Trihydroxy-trimethoxy(iso)flavone isomer 3	C18H16O8	32,63		359,07670
Dimethoxy-trihydroxy(iso)flavones	C17H14O7	32,85		329,06613
Dihydroxy-tetramethoxy(iso)flavones	C19H18O8	33,26		373,09235
Pinellic acid	C18H34O5	33,61		329,23280

## 4 Discussion

This study appraised the nutritional profile of *L. spathulatum* leaves aiming to evaluate its suitability for human consumption. Its moisture level was like the values reported for other halophytes species, such as *Polygonum maritimum* L. cultivated with saline water containing up to 100 mM of sodium chloride (NaCl) (sea knotgrass, 70 – 80%; Rodrigues et al., 2019) and *L. algarvense* Erben cultivated in greenhouse conditions and irrigated with freshwater (79.8%; Rodrigues et al., 2020). However, moisture was lower than the values reported for edible halophytes characterized by its succulence, such as *Sarcocornia* and *Salicornia* species, which moisture levels are usually higher than 85% (Custódio et al., 2021), and of some common vegetables, including *Lactuca sativa* L. (lettuce, 94.7%) (Custódio et al., 2021; USDA, 2021). A high moisture content is usually related to a higher tendency for food spoilage, as observed for example in lettuce (Barg et al., 2008; Kyere et al., 2020), therefore having a high influence on the product shelf life and in the consumers’ acceptance of a product. Therefore, *L. spathulatum* with a lower moisture level than other common edible succulent halophytes may result in a greater consumer acceptability.

The ash content of a plant biomass is related to its total mineral level. Halophytes thrive in saline conditions, have a high capacity to absorb and retain minerals without toxic effects to the plant, and therefore, usually have higher ash contents than glycophyte plants (Borah et al., 2008; Díaz et al., 2013). The ash content of *L. spathulatum* similar to that of the halophyte *Cladium mariscus* L. (Pohl.) It was however lower than the ash levels of related species, including *L. axillare* (Forssk.) Kuntze (Al-Easa, 2003) and *L. pruinosum* (L.) Chaz (El-Amier and Ejgholi), and also than other edible halophytes, including *Sarcocornia* and *Salicornia* Custódio et al., 2021). The ash level of *L. spathulatum* was however higher than that of lettuce cultivated in hydroponics and in the soil (Lei and Engeseth, 2021). Such differences may be dependant on the species and/or on the mineral level of the soils from which the plants were collected.

Halophytes usually have a high content in dietary fibre (Díaz et al., 2013). In this work, NDF was determined to estimate the quantities of fibres including cellulose, hemicellulose, and lignin, and also cutin (Dhingra et al., 2012). While being normally used to appraise feed quality, NDF is considered a valuable tool to estimate the insoluble portion of dietary fibre in food (McDougall et al., 2009; Dhingra et al., 2012). The level of NDF of *L. spathulatum* leaves is higher than that reported for other vegetables, including *Lens culinaris* Medik (McDougall et al., 2009; Dhingra et al., 2012), and other edible halophytes, such as *Sarcocornia perennis subsp. alpini* (Mill.)and *Salicornia ramosissima* J.Woods (Barreira et al., 2017). It was however lower than *Bassia hyssopipifolia* (Pall.) Kuntze (Díaz et al., 2013). Our results suggest that *L. spathulatum* is a good source of fiber, which has relevant health advantages including prevention of cardiovascular diseases and diabetes, besides contributing to weight loss, due to its low caloric content (Whelton et al., 2005; Yao et al., 2014).

The crude protein of *L. spathulatum* was as expected low but higher than that of *L. axillare*, *Sarcocornia* and *Salicornia* (Custódio et al., 2021), and *C. mariscus* Oliveira-Alves et al., 2021). It was however lower than other *Limonium* species, such as *L. pruinosum* and other common vegetables, including lettuce and spinach (USDA, 2021), thus suggesting that the consumption of *L. spathulatum* can contribute to a higher input of protein that these latter species.

Similar to protein, the crude fat content of *L. spathulatum* was also low, and lower than the levels detected in other *Limonium* species, such as *L. pruinosum* (0.92%) and *L. axillare*, and also than other edible halophytes, including *S. perennis perennis* and *S. perennis alpini* (Akyol et al., 2020), and some common vegetables, such as raw lettuce (*Lactuca sativa* var. logifolia and spinach (USDA, 2021). Moreover, *L. spathulatum* also had low levels of carbohydrates level, which resulted in a low energetic value (33.7kcal/100 g, dw, corresponding to 7.49 kcal/100 g, fw), lower than the values reported for common vegetables, includings lettuce (20 kcal/100 g, fw), spinach (27 kcal/100 g, fw) (USDA, 2021) and *Salicornia bigelovii* (3.8 MJ kg^−1^, dw, corresponding to 20.17 kcal/100 g, fw) (Díaz et al., 2013). Such a low energy value, combined with the low-fat and carbohydrates content, suggests that consuming *L. spathulatum* leaves can contribute to weight loss, and therefore, to prevent relevant non communicable diseases.

Dietary minerals have vital roles in the human body, including bone formation and muscle function (Gharibzahedi and Jafari, 2017), and can be obtained from different food sources, including vegetables, fruits, and animal products. Halophytes have a high capacity to accumulate minerals without toxicity and are therefore indicated as very interesting sources of such elements. In this work, the most abundant macroelements detected in *L. spathulatum* leaves were Cl^-^, Ca and Na, while the most abundant microelements were Fe and Zn. Although Cl^-^ was previously considered harmful to conventional crops due to its impairment effects on nitrate ( ${\text{NO}}_{3}^{–}$ ) nutrition and consequent crop yield reduction, new findings show its beneficial properties, including improvement of the overall plant growth, tissue water balance, plant water relations, photosynthetic performance, and water-use efficiency (Raven, 2016; Rosales Miguel et al., 2020). Most glycophytes contain 1 - 20 mg Cl^−^ g (dw) (Marschner, 2011), while in halophytes Cl^−^ is only toxic at concentrations higher than 50 mg/g (dw) (Geilfus, 2018), which is a higher value than that detected in *L. spathulatum*.

The Na content of *L. spathulatum* leaves were lower than the level detected in the same species collected in different locations, in Tunisia (Souid et al., 2019), and than the values reported for different edible halophytes, such as *Sarcocornia* and *Salicornia* species (Custódio et al., 2021). It was however higher than the levels detected in the leaves of drought-resistant amaranth (Sarker et al., 2022a), A. tricolor (Sarker and Oba, 2020a) and the leaves of *Cladium. mariscus* Oliveira-Alves et al., 2021), and in the range of the levels reported for common green vegetables, including (Kim et al., 2016) and seaweed (El-Said and El-Sikaily, 2012). According to the World Health Organization (WHO), the Na daily intake should not exceed 2 g. Therefore, to achieve the maximum daily intake of Na it would be necessary to consume as much as 553.08 g of fresh leaves of *L. spathulatum*.

The Ca concentration detected in *L.* spathulatum was higher than those of the leaves of danta (Sarker et al., 2022b), A. lividus (Sarker et al., 2022c), stem amaranth (Sarker et al., 2022d), *Salicornia perennis*, *S. ambigua*, and *S. neii* (Bertin et al., 2014; Riquelme et al., 2016; Barreira et al., 2017), but lower than the Ca level *S. fruticosa* (Castañeda-Loaiza et al., 2020a). *Limonium spathulatum* leaves can be considered good source of Ca when compared with vegetables considered rich sources of this element, such as kale, (USDA, 2021). The daily recommended dietary allowances (RDA) for Ca are age and country dependent (Rose and Strombom, 2019), and usually peak in the adolescence (1300 mg) and in the elderly (1000 – 1200 mg) (Rose and Strombom, 2019). The consumption of 100 g of fresh *L. spathulatum* leaves would cover 38 and 29% of the RDA for the elderly and adolescents, respectively. The intake of vegetables rich in Ca is especially important in vegetarians and vegans, where no dairy products are consumed. While absorption of Ca from vegetables is often better than from dairy products, bioavailability issues may arise related with the oxalate levels of plant tissues, since Ca absorption is inversely proportional to the oxalic acid content of the food (Rose and Strombom, 2019). Therefore, future studies should consider determining the oxalate levels of *L. spathulatum* leaves.

Iron was the major micro element in L. *spathulatum*, in similar or lower levels than those detected in *Sarcocornia* species (Riquelme et al., 2016; Barreira et al., 2017). It was however higher than and in Fe rich vegetables, such as parsley (*Petroselinum crispum* (Mill.) Fuss) (USDA, 2021). Therefore, consuming 84 g and 191 g of fresh *L. spathulatum* could contribute to fulfill the recommended daily Fe intake of 8 - 18 mg/day for adults (Trumbo et al., 2001). The Zn levels of *L. spathulatum* were in the range than those in different *Sarcocornia* and *Salicornia* species (Custódio et al., 2021). These were however higher than and spinach (USDA, 2021). The consumption of 1.4 and 1.9 g of fresh *L. spathulatum* could contribute to fulfill the recommended daily Zn intake of 8 - 11 mg/day for adults (Trumbo et al., 2001).

The iodine level of *L. spathulatum* was lower than that found in some edible halophytes, such as *Crithmum. maritimum*, grown in a hydroponic system (Sarroua et al., 2019) and *Inula crithmoides* L. cultivated in a controlled environments under irrigation with different salinities *(* Zurayk and Baalbaki, 1996). It was however higher than lettuce and asparagus (*Asparagus officinalis* L.) (WHO, 2018), and therefore, could be an interesting source of iodine, when compared with common vegetables, especially for pregnant woman.

Halophytes can accumulate toxic metals, including Pb and Cd, when growing in contaminated soils (Caetano et al., 2008). However, the accumulation of such elements generally occurs in the roots, since its translocation to aboveground organs is limited, as observed in different halophytic species, such as *S. fruticosa*, *S. ramosissima* and *A. macrostachyum* (Caetano et al., 2008; Moreira da Silva, 2008; Redondo-Gómez et al., 2010). In this work, Pb and Cd, were not detected in the leaves of *L. spathulatum*. Some other molecules exhibit toxicity and/or antinutrient activity may be present in halophytes. This is the case of tannins, phytic acid, trypsin and alpha-amylase inhibitors which are considered antinutritional factors since they might interfere with the bioavailability and/or digestibility of some nutrients, including proteins and minerals (Samtiya et al., 2020). In this work, the extracts of *L. spathulatum* were phytic acid free and presented a high capacity to inhibit trypsin, but reduced α-amylase inhibition, when tested at 1 mg/mL.

In this work, the antioxidant potential of *L. spathulatum* leaves was evaluated by different *in vitro* methods, covering different mechanisms of action, namely those involving free radicals and metal ions. The ethanol and the hydroethanolic extracts had in general the highest capacity to scavenge free radicals when compared to water extracts, thus suggesting that such extracts contain primary antioxidant compounds with the capacity to neutralize free radicals and prevent the initiation and propagation of oxidative chain reactions (Loganayaki and Manian, 2010). Such activity was similar or higher than that of the tested standard (BHT), which is one of the most used synthetic antioxidant additives to food stuffs In general, *Limonium* species are acknowledged as sources of strong antioxidants. For example, a free radical scavenging activity guided fractionation of a methanol root extract and obtained fractions of *L. brasiliense* Kuntze resulted in the isolation of five active antioxidant compounds, namely gallic acid, epigallocatechin 3-*O*-gallate, epigallocatechin, gallocatechin and myricetin 3-*O*-α-rhamnoside (myricitrin) (Murray et al., 2004). Myricitrin exhibits relevant antioxidant properties, with stronger free radical scavenging activity than other flavonol rhamnosides or quercetin (Wu et al., 2008); all detected in the *L. spathulatum* extracts. Methanol leaf extracts of *L. algarvense* also had a strong capacity to scavenge the DPPH radical, with an EC_50_ value of 0.54 mg/mL (Rodrigues et al., 2015), although less effective than *L. spathulatum*.

The strong antioxidant potential of *L. spathulatum* is most probably related with its high content in polyphenolic compounds, since such molecules are recognized antioxidant agents (Granato et al., 2018; Stanković et al., 2019).

Since a high antioxidant activity was obtained in the *in vitro* assays, *L. spathulatum* was evaluated for the first time for their ability to reduce lipid peroxidation in porcine brain cell membranes (TBARS) and oxidative hemolysis of sheep erythrocytes **(**OxHLIA). Such assays are appropriate *ex vivo* models for evaluating inhibition of lipid peroxidation by the presence of antioxidants (Takebayashi et al., 2009; Takebayashi et al., 2012). Similar to the observed in the free radical and metal-based assays, the upmost activity was observed after the application of the hydroethanolic and ethanol extracts, which may be related with the highest levels of polyphenolics and flavonoids detected in such extracts, as stated before. A relevant inhibition of lipidic peroxidation was also detected in a water extract from leaves of *L. algarvense* (Rodrigues et al., 2015). Lipids are highly vulnerable to peroxidation, which is linked with the onset of several degenerative disorders, including cardiovascular (Gianazza et al., 2021) and neurodegenerative diseases (Angelova et al., 2021). In addition, lipid peroxidation alters the composition, structure, and function of the lipids present in cellular membranes, that may result in DNA and proteins damage. The use of natural products from *limonium species such as L. spathulatum* capable to decrease cellular lipid peroxidation is therefore considered an important therapeutical tool to prevent the occurrence of degenerative and chronic disorders linked to oxidative stress. There is an increasing interest in the use of these natural extracts to improve foodstuff stability (Da Silva et al., 2021). The high activity detected in the ethanolic extract may be related with its higher level of total polyphenolic compounds, while the activity of the ethanol extract is most probably related with its richness in flavonoids.

This hypothesis was conformed by the study of TPC and TFC and the identification of their individual compunds in the most active ethanol extracts of *L. spathulatum*. TPC of *L. spathulatum* leaves of all extracts were greater than the leaves of drought-tolerant leafy vegetable amaranth (Sarker and Oba, 2020b), *Amaranthus gangeticus* (Sarker and Oba, 2020a). Such levels are higher when compared to other medicinal halophytes species with confirmed pharmacological properties such as *Limoniastrum monopetalum* (L.) Boiss, Trabelsi et al., 2012), *Tamarix gallica* L. and *Mesembryanthemum edule* L. (syn. *Carpobrotus edulis* L.) (Ksouri et al., 2008), and also higher than the levels detected in water extracts made from different medicinal herbs and spices, *Rosmarinus officinalis* L., *Salvia officinalis* L., *Thymus vulgaris* L. and *Origanum vulgare* L. (Ulewicz-Magulska and Wesolowski, 2019). The TPC of *L. spathulatum* was similar than that detected in a methanol extract of the same species from Algeria (Mazouz et al., 2020), but higher than that detected in a ethanol extract from *L. boitardii* (Sefi et al., 2021), and of a methanol extract from leaves from *L. algarvense* (Rodrigues et al., 2015). In plants, phenols are responsible for pigmentation (Sarker and Oba, 2020a; Sarker and Oba, 2021) and astringency, serve as protective agents against abiotic (*e.g.*,UV light), and biotic (*e.g.*, parasites and insects) stress (Caleja et al., 2017; Durazzo et al., 2019). Such molecules also have important human health implications, since they exhibit relevant health improvement properties, including antioxidant, anti-diabetic, anti-inflammatory and anti-tumor (Albuquerque et al., 2020; Diasa et al., 2021).

More interestingly, flavonoids peaked in the ethanol extract, similar to the total flavonoids found in a methanol extract from *L. algarvense* (Rodrigues et al., 2015), but in lower amounts than those detected in a hydroethanolic leaf extract from *L. boitardii* (Sefi et al., 2021). Such differences are highly dependent on several factors, includings the type of extraction used, plant species, as well as biotic and abiotic stresses (Do et al., 2014; Karoune et al., 2015; Cujic et al., 2016; Bakhouche et al., 2021). Flavonoids exhibit important biological properties potentially associated with multiple health benefits to the antioxidant system of the human body. They are also considered as an important element in dietary supplements, pharmaceutical, medicinal and commercial applications. (Panche et al., 2016; Castañeda-Loaiza et al, 2020b).

The major molecules identified in the ethanolic extracts were, mainly hydroxybenzoic acids (gallic, syringic), hydroxycinnamic acids (caffeic, coumaric, ferulic acids) and flavonoids (catechin, epigallocatechin gallate and naringin). Some compounds were already been described in a related species, *L. boitardii* (Sefi et al., 2021), namely gallic acid,epigallocatechin-3-*O*-gallate (Teatannin II), rutin (quercetin-3-*O*-rutinoside), myricetin (3,3’,4’,5,5’,7-Hexahydroxyflavone), and quercetin (3,3’,4’,5,7-Pentahydroxyflavone). Quinic acid is a chlorogenic acid metabolite, and was already reported in methanol extracts from aerial parts of *L. tubiflorum* (Delile) Kuntze var tubiflorum (El-Kousy et al., 2021). Quinic acid is an organic acid mediating the ‘‘shikimate pathway’’ (shikimic acid pathway), which is a chief aromatic amino acid synthesis metabolic route exclusive to plants and microorganisms resulting in the formation of tryptophan (TRP), tyrosine (TYR), and phenylalanine (PHE) (Averesch and Krömer, 2018). Quinic acid has important biological properties, including antioxidant (Bursal et al., 2018), antimicrobial (Lu et al., 2021; Bai et al., 2022) and anti HIV-1 (Yazdi et al., 2019), and is a building block for the synthesis of several valuable secondary compounds, including coumaroyl and caffeoylquinic acid derivatives with significant biological activity in several drug-target areas (Cheynier et al., 2012). A related compound of quinic acid, shikimic acid, was also detected in the ethanolic extract from *L. spathulatum*, is also key intermediate of the ‘‘shikimate pathway’’ and has a high pharmaceutical importance, such as being a precursor for the synthesis of oseltamivir (Tamiflu), the only drug against avian flu caused by the H5N1 virus (Quiroz et al., 2014; Bai et al., 2022). Myricetin-*O*-galloylhexoside, myricetin-*O*-(di-*O*-acetyl)rhamnoside isomer 1, and myricetin-*O*-(di-*O*-acetyl)rhamnoside isomer 2, previously identified in ethanol extracts from aerial parts of *L. caspium* (Willd) (Gadetskaya et al., 2015), and isolated from *L. sinuatum* (L.) Mill and *L. meyeri* (Boiss.) Kuntze (Ross, 1984; Movsumov and Garaev, 2005), while Myricetin-3-*O*-rutinoside was previously identified in *L. algarvense’s* water extracts (Rodrigues et al., 2021). Myricetin, and its derivatives, exhibit important biological properties, including antioxidant, anticarcinogenic, antiviral and antimicrobial (Baysal et al., 2021; Sinan et al., 2021). Prodelphinidin A gallate and ethyl gallate were previously detected in *L. bondueli* organs (Breant et al., 2010). Chlorogenic acid, gallic acid and rutin were identified in the shoot extracts of *L. delicatulum* (Baysal et al., 2021). High amounts of epigallocatechin gallate, phlorizin, phloretin and quercetin were also detected in aqueous extracts of *L. contortirameum* and *L. virgatum* (Foddai et al., 2014), while tannic acid and hyperoside were quantified in high levels in the ethyl acetate fractions of aerial organs *L. effusum* and *L. sinuatum* (Baysal et al., 2021).

In conclusion, the leaves of *L. spathulatum* collected from Tunisian sea cliffs were good source of minerals and fibers useful in the human diet for attaining nutritional sufficiency. The high *in vitro* and *ex vivo* antioxidant activities associated with high phenolics and favonoids contents and compounds suggest the possibility to use extracts of *L. spathulatum* in herbal products with the aim of improving general health and well-being, and/or as food additives for preventing lipid oxidation of lipid-rich foods.

## Data availability statement

The raw data supporting the conclusions of this article will be made available by the authors, without undue reservation.

## Author contributions

SY: Conceptualization, Data curation, Writing-Original draft preparation, Writing-Review and Editing, Figures and tables. LC: Conceptualization, Data curation, Writing-Original draft preparation, Writing-Review and Editing, Supervision. MR, CP: Data curation, Writing-Original draft preparation. RC, JP, LB, JJ and ZC: Data curation. KH: Conceptualization, Writing-Original draft preparation, Writing-Review and Editing, Supervision. All authors contributed to the article and approved the submitted version.

## Acknowledgments

The authors are grateful to the Tunisian Ministry of Higher Education and Scientific Research and the Foundation for Science and Technology (FCT, Portugal) for financial supports. This work was also made under the frame of the project HaloFarMs, which is part of the Partnership on Research and Innovation in the Mediterranean Area (PRIMA). S.Y. was supported by the University of Tunis El Manar. L.C. was supported by the FCT Scientific Employment Stimulus (CEEC-IND/00425/2017). M.J.R was supported through the FCT programme contract (UIDP/04326/2020). S.Y and K.B.H are thankfull to Dr. Abidi S. from INRAT (Tunisia) for technical assistance in fiber analysis.

## Conflict of interest

The authors declare that the research was conducted in the absence of any commercial or financial relationships that could be construed as a potential conflict of interest.

## Publisher’s note

All claims expressed in this article are solely those of the authors and do not necessarily represent those of their affiliated organizations, or those of the publisher, the editors and the reviewers. Any product that may be evaluated in this article, or claim that may be made by its manufacturer, is not guaranteed or endorsed by the publisher.
